# Supplementary material for: Effects of infectious disease consultation and antimicrobial stewardship program at a Japanese cancer center: An interrupted time-series analysis
Source: PLoS One. 2022 Jan 25;17(1):e0263095. doi: 10.1371/journal.pone.0263095 (PMC8789186; doi:10.1371/journal.pone.0263095)
Supplement: S1 Fig — Each dot refers to the incidence of CRE per 1000 patients each month and the slope is based on linear regression in the two phases. The explanation of each phase is as follows: Phase 1 (antimicrobial notification by the infection control team from April 1, 2018, to March 31, 2020); Phase 2 (establishing an infectious disease [ID] consultation service and implementation of the Antimicrobial Stewardship Program [ASP] from April 1, 2020, to March 31, 2021). The number of isolated samples in 2018, 2019, and 2020 was 10, 5, and 8, respectively. There was no increase in the trend of the monthly incidence of CRE (coefficient: 0.012; 95% confidence interval [CI]: −0.001 to 0.03, p = 0.09) or a change in its level after the implementation of the Antimicrobial Stewardship Program (coefficient: −0.02; 95% CI: −0.12 to 0.09, p = 0.78). (DOC) [file pone.0263095.s001.doc]

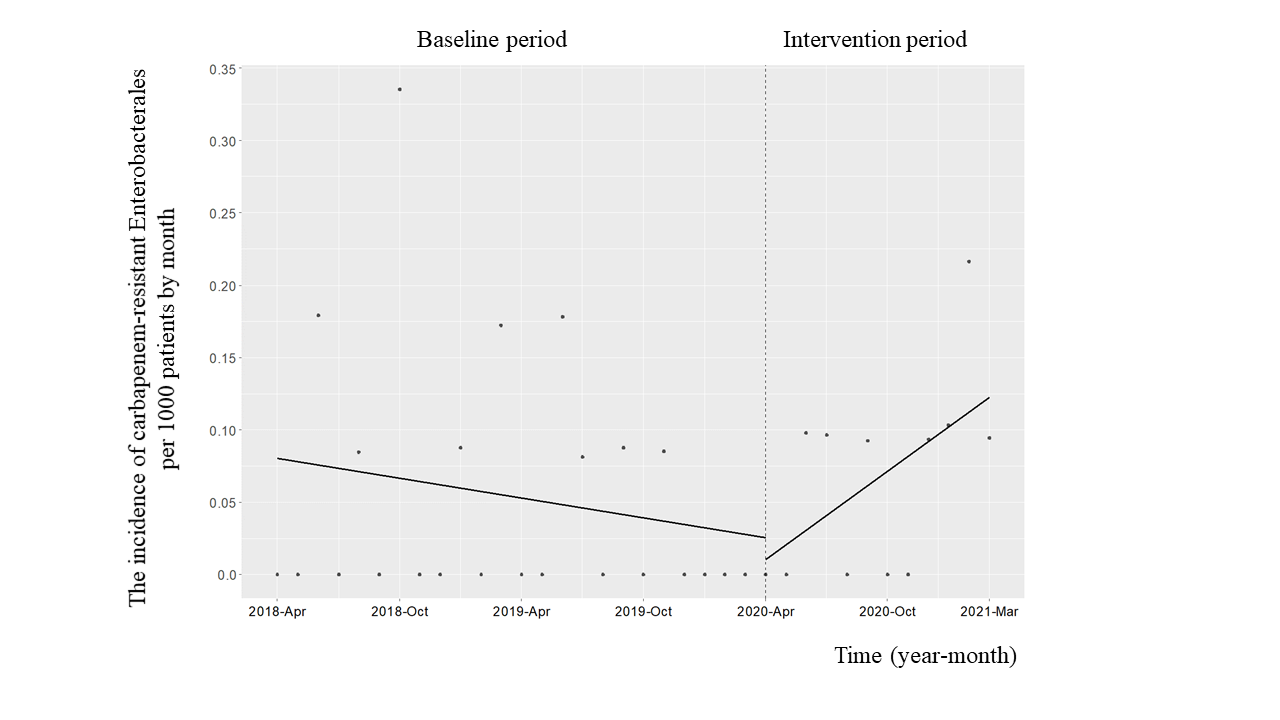
**S1 Fig.** **Trends of the incidence of** **carbapenem-resistant *Enterobacteriaceae* (CRE) per 1000 patients, by month,** **during Phase 2 of the intervention period.** Each dot refers to the incidence of CRE per 1000 patients each month and the slope is based on linear regression in the two phases. The explanation of each phase is as follows: Phase 1 (antimicrobial notification by the infection control team from April 1, 2018, to March 31, 2020); Phase 2 (establishing an infectious disease [ID] consultation service and implementation of the Antimicrobial Stewardship Program [ASP] from April 1, 2020, to March 31, 2021). The number of isolated samples in 2018, 2019, and 2020 was 10, 5, and 8, respectively. There was no increase in the trend of the monthly incidence of CRE (coefficient: 0.012; 95% confidence interval [CI]: −0.001 to 0.03, *p*=0.09) or a change in its level after the implementation of the Antimicrobial Stewardship Program (coefficient: −0.02; 95% CI: −0.12 to 0.09, *p*=0.78).
