# Supplementary material for: Effects of infectious disease consultation and antimicrobial stewardship program at a Japanese cancer center: An interrupted time-series analysis
Source: PLoS One. 2022 Jan 25;17(1):e0263095. doi: 10.1371/journal.pone.0263095 (PMC8789186; doi:10.1371/journal.pone.0263095)
Supplement: S5 Fig — Each dot refers to the in-hospital mortality each month and the slope is based on linear regression in the two phases. The explanation of each phase is as follows: Phase 1 (antimicrobial notification by the infection control team from April 1, 2018, to March 31, 2020); Phase 2 (establishing an infectious disease [ID] consultation service and implementation of the Antimicrobial Stewardship Program [ASP] from April 1, 2020, to March 31, 2021). There was no significant change in the trend of in-hospital mortality (coefficient: −0.10; 95% confidence interval [CI]: −0.23 to 0.02, p = 0.122) or in its level (coefficient: −0.36; 95% CI: −1.33 to 0.60, p = 0.47). (DOC) [file pone.0263095.s005.doc]

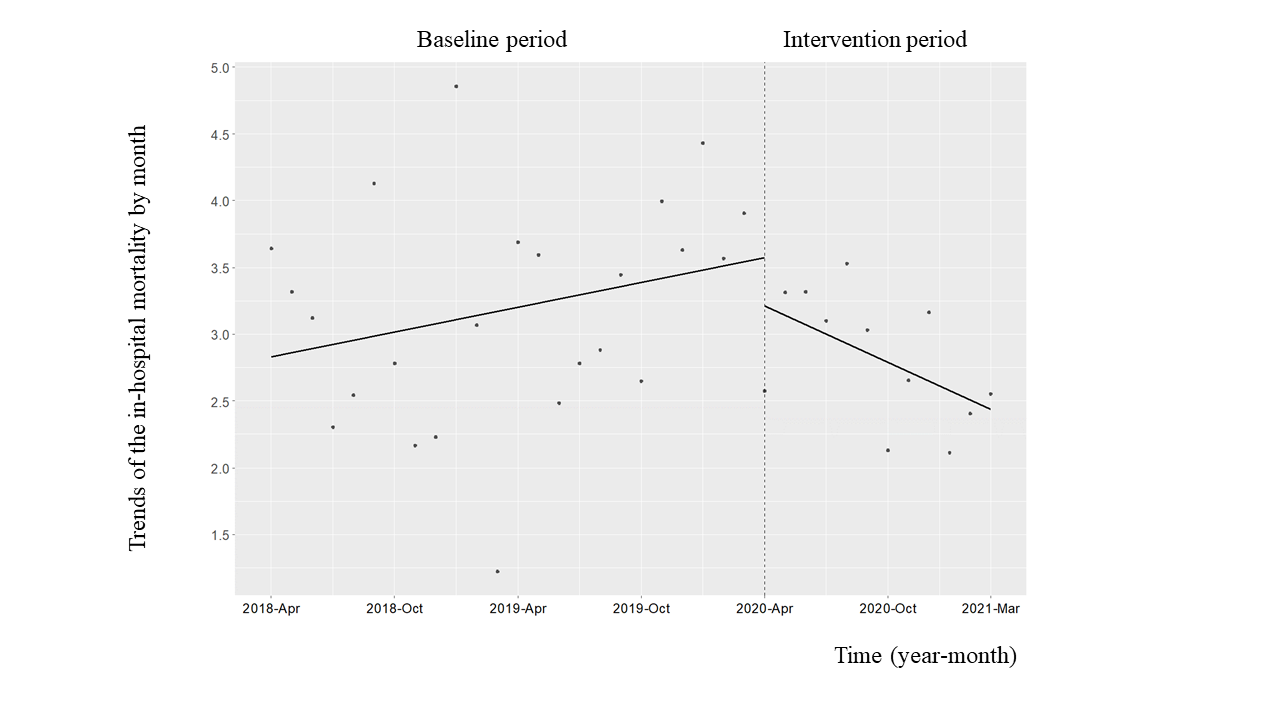
**S5 Fig.** **Trends of the in-hospital mortality by month during Phase 2 of the intervention period.** Each dot refers to the in-hospital mortality each month and the slope is based on linear regression in the two phases. The explanation of each phase is as follows: Phase 1 (antimicrobial notification by the infection control team from April 1, 2018, to March 31, 2020); Phase 2 (establishing an infectious disease [ID] consultation service and implementation of the Antimicrobial Stewardship Program [ASP] from April 1, 2020, to March 31, 2021). There was no significant change in the trend of in-hospital mortality (coefficient: −0.10; 95% confidence interval [CI]: −0.23 to 0.02, *p*=0.122) or in its level (coefficient: −0.36; 95% CI: −1.33 to 0.60, *p*=0.47).
