# Supplementary material for: Effects of infectious disease consultation and antimicrobial stewardship program at a Japanese cancer center: An interrupted time-series analysis
Source: PLoS One. 2022 Jan 25;17(1):e0263095. doi: 10.1371/journal.pone.0263095 (PMC8789186; doi:10.1371/journal.pone.0263095)
Supplement: S6 Fig — Each dot refers to the length of hospital stay each month and the slope is based on linear regression in the two phases. The explanation of each phase is as follows: Phase 1 (antimicrobial notification by the infection control team from April 1, 2018, to March 31, 2020); Phase 2 (establishing an infectious disease [ID] consultation service and implementation of the Antimicrobial Stewardship Program [ASP] from April 1, 2020, to March 31, 2021). There was no significant change in the trend of the length of hospital stay (coefficient: −0.06; 95% confidence interval [CI]: −0.13 to 0.01, p = 0.12) or its level (coefficient: −0.49; 95% CI: −1.1 to 0.08, p = 0.10). (DOC) [file pone.0263095.s006.doc]

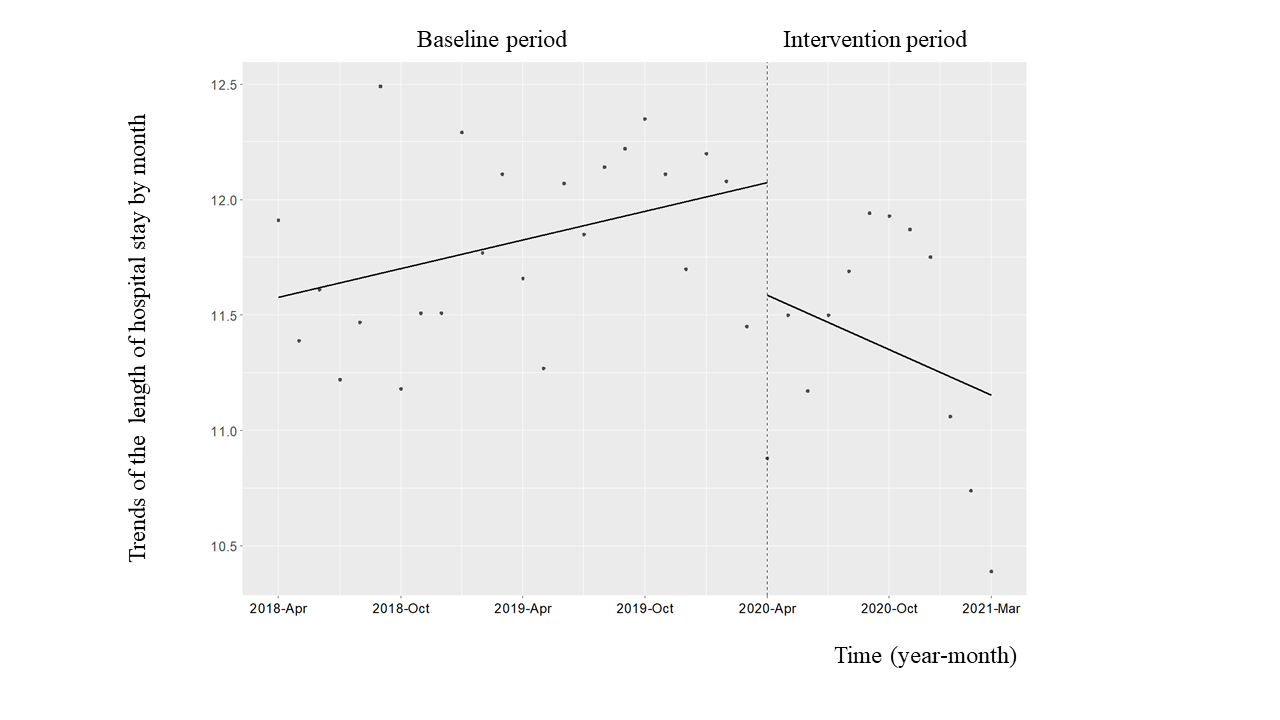
**S6 Fig. Trends of the length of hospital stay by month during Phase 2 of the intervention period**. Each dot refers to the length of hospital stay each month and the slope is based on linear regression in the two phases. The explanation of each phase is as follows: Phase 1 (antimicrobial notification by the infection control team from April 1, 2018, to March 31, 2020); Phase 2 (establishing an infectious disease [ID] consultation service and implementation of the Antimicrobial Stewardship Program [ASP] from April 1, 2020, to March 31, 2021). There was no significant change in the trend of the length of hospital stay (coefficient: −0.06; 95% confidence interval [CI]: −0.13 to 0.01, *p*=0.12) or its level (coefficient: −0.49; 95% CI: −1.1 to 0.08, *p*=0.10).
