# Supplementary material for: Effects of infectious disease consultation and antimicrobial stewardship program at a Japanese cancer center: An interrupted time-series analysis
Source: PLoS One. 2022 Jan 25;17(1):e0263095. doi: 10.1371/journal.pone.0263095 (PMC8789186; doi:10.1371/journal.pone.0263095)
Supplement: Attachment — Submitted filename: Response to Reviewers_2.docx [file pone.0263095.s008.docx]

**Our point-by-point responses to the comments and suggestions of Reviewer 2 are listed below.**

We thank the reviewer for reviewing our manuscript and for the insightful comments that have helped us significantly improve our manuscript. Please note that our changes in the manuscript are highlighted in yellow.

**Comment 1:**

In the present study, authors defined the usage of antibiotics with the DOT per month and per 100 patient-days. However, I am wondering whether the amounts or types (e.g., oral or IV) of antibiotics were almost identical over time and over patients. Because if the variations in the used amount of antibiotics are not negligible, the calculation using the DOT may provide some biases, although it still can act as an indirect indicator. If so, I would like to suggest introducing the concept of the defined daily dose (DDD) to fully consider the amount of antibiotics in each patient.

**Response:**

We wish to thank the reviewer for these important comments. We calculated the AUD using DDD in addition to the DOT data, following the reviewer's suggestion, and performed additional analysis. The results of the analysis using AUD were the same as the results of the analysis using DOT. Accordingly, we have added sentences as follows:

“The results of these analyses of antimicrobial use are consistent with the DOT-based data based on antimicrobial use density (AUD).” (Abstract: Page 2, Lines 39-40).

“Antimicrobial use density (AUD) for carbapenem, three antipseudomonal agents, four narrow-spectrum antimicrobials

AUD was also calculated to normalize the amount of each antimicrobial consumption. AUD was expressed as defined daily dose (DDD) per 100 patient-days for individual antimicrobial agent (AUD ＝antimicrobial consumption/DDD×100/patient-days). DDD per 100 inpatient-days for each drug or drug category prescribed monthly was calculated following the 2021 World Health Organization (WHO) Anatomical Therapeutic Chemical (ATC) classification system [14]. AUDs were calculated for carbapenem (CAR-AUD; for imipenem–cilastatin, meropenem, and doripenem); three antipseudomonal agents (piperacillin–tazobactam, cefepime, and cefozopran); and four narrow-spectrum antimicrobials (ampicillin, ampicillin–sulbactam, cefazolin, and cefmetazole).“ (Methods section, Pages 7-8, Lines: 147-156).

“To demonstrate the effect of each intervention on CAR-DOT, CAR-AUD, the DOT, and AUD for the three antipseudomonal agents (piperacillin–tazobactam, cefepime, and cefozopran) and four narrow-spectrum antibiotics (ampicillin, ampicillin–sulbactam, cefazolin, and cefmetazole) were ascertained. Furthermore, we carried out a segmented regression analysis of interrupted time-series studies for the two periods (Phase 1: April 1, 2018 to March 31, 2020; Phase 2: April 1, 2020 to March 31, 2021), when the DOT data were available.” (Statistical analysis, Page 9, Lines: 201-205).

“The trend in the CAR-AUD decreased along with its level (trend change, coefficient: -0.10; 95% CI: -0.15 to 0.05, p<0.001; change in level, coefficient: -0.60; 95% CI: -0.98 to -0.23, p<0.001).” (Results, Page 10, Lines: 233-234).

“The level of the monthly AUD for the three antipseudomonal agents did not decrease (coefficient: -0.01; 95% CI: -0.75 to 0.73, p=0.98), although its trend reduced (coefficient: -0.12; 95% CI: -0.22 to -0.031, p=0.01).” (Results, Page11, Lines: 250-252).

“The trend in the monthly AUD of the four narrow-spectrum antibiotics increased along with its level (trend change, coefficient: 0.43; 95% CI: 0.36 to 0.51, p<0.001; change in level, coefficient: 1.45; 95% CI: 0.85 to 2.04, p<0.001).” (Results, Page 12, Lines: 268-270).

“The results of the analysis between antimicrobial use using AUD and DOT were consistent.” (Discussion, Page 16, Lines: 374-375).

**Comment 2:**

In the interrupted time-series (ITS) analyses, changes in the shape indicate the immediate effect of intervention, while the sustained effect can be represented by changes in the slope. Thus, although authors asserted that the estimated level is expected to decrease in the long-term observation (L340), the duration of observation will not affect the significance of changes in the shape. If authors tried to emphasize the expected sustained decreases in the DOT based on the significant decreases in the slope, I would like to suggest that authors can rephrase the sentence for the naïve readers.

**Response:**

We wish to thank the reviewer for these insightful comments. Accordingly, we have added the following sentences:

“The levels of the three antipseudomonal agents did not decrease significantly, but the results suggest that a decrease can be expected in the future if significant decrease in trend is sustained” (Discussion, Page16, Lines: 366-368).

**Comment 3:**

Furthermore, according to the figures (especially in Figure 1 and Figure 3), the trends of DOT were started to be mainly changed from the late 2019, regardless of the start of intervention was April 2020. In addition, if authors think the start point of visual impact of countermeasures can be delayed (e.g., delayed by a certain period of time-gap from the official start time of intervention), I would like to suggest considering a sensitivity analysis by varying the start of intervention time (adjusted start time considering the time-gap), since it may change the estimated changes in the level.

**Response:**

We wish to thank the reviewer for these astute comments. The reason for the change was assumed to be due to the influence of an approximately one-hour lecture of an external infectious disease specialist on appropriate antimicrobial use for healthcare professionals on February 19, 2019. Sensitivity analysis was not performed because apparently, our active intervention was from April 2020. Accordingly, we have added the sentences as follows:

“Our interventions began in April 2020, but changes have been occurring in the use of carbapenems and the three anti-pseudomonal agents since late 2019. This was partly due to the impact of a one-hour lecture on the appropriate use of antimicrobials to healthcare providers by an external ID expert on February 19, 2019.” (Discussion, Page 17, Lines 405-407).
